# Distinct white matter microstructural abnormalities and extracellular water increases relate to cognitive impairment in Alzheimer’s disease with and without cerebrovascular disease

**DOI:** 10.1186/s13195-017-0292-4

**Published:** 2017-08-17

**Authors:** Fang Ji, Ofer Pasternak, Siwei Liu, Yng Miin Loke, Boon Linn Choo, Saima Hilal, Xin Xu, Mohammad Kamran Ikram, Narayanaswamy Venketasubramanian, Christopher Li-Hsian Chen, Juan Zhou

**Affiliations:** 1 Center for Cognitive Neuroscience, Neuroscience and Behavioral Disorders Program, Duke-NUS Medical School, 8 College Road, 06-15, Singapore, 169857 Singapore; 2 Department of Psychiatry, Brigham and Women’s Hospital, Harvard Medical School, Boston, MA USA; 3 Department of Radiology, Brigham and Women’s Hospital, Harvard Medical School, Boston, MA USA; 4 Department of Pharmacology, Clinical Research Centre, National University Health System, National University of Singapore, Singapore, 117600 Singapore; 5 Memory Aging & Cognition Centre, National University Health System, National University of Singapore, Singapore, Singapore; 6 Raffles Neuroscience Centre, Raffles Hospital, Singapore, Singapore; 7 Clinical Imaging Research Centre, Agency for Science, Technology and Research, Singapore, Singapore

**Keywords:** Alzheimer’s disease, Cerebrovascular disease, Diffusion tensor imaging, Free water imaging, Extracellular water, Vascular damage, Cognitive impairment

## Abstract

**Background:**

Mixed vascular and neurodegenerative dementia, such as Alzheimer’s disease (AD) with concomitant cerebrovascular disease, has emerged as the leading cause of age-related cognitive impairment. The brain white matter (WM) microstructural changes in neurodegeneration well-documented by diffusion tensor imaging (DTI) can originate from brain tissue or extracellular free water changes. The differential microstructural and free water changes in AD with and without cerebrovascular disease, especially in normal-appearing WM, remain largely unknown. To cover these gaps, we aimed to characterize the WM free water and tissue microstructural changes in AD and mixed dementia as well as their associations with cognition using a novel free water imaging method.

**Methods:**

We compared WM free water and free water-corrected DTI measures as well as white matter hyperintensity (WMH) in patients with AD with and without cerebrovascular disease, patients with vascular dementia, and age-matched healthy control subjects.

**Results:**

The cerebrovascular disease groups had higher free water than the non-cerebrovascular disease groups. Importantly, besides the cerebrovascular disease groups, patients with AD without cerebrovascular disease also had increased free water in normal-appearing WM compared with healthy control subjects, reflecting mild vascular damage. Such free water increases in WM or normal-appearing WM (but not WMH) contributed to dementia severity. Whole-brain voxel-wise analysis revealed a close association between widespread free water increases and poorer attention, executive functioning, visual construction, and motor performance, whereas only left hemispheric free water increases were related to language deficits. Moreover, compared with the original DTI metrics, the free water-corrected DTI metric revealed tissue damage-specific (frontal and occipital) microstructural differences between the cerebrovascular disease and non-cerebrovascular disease groups. In contrast to both lobar and subcortical/brainstem free water increases, only focal lobar microstructural damage was associated with poorer cognitive performance.

**Conclusions:**

Our findings suggest that free water analysis isolates probable mild vascular damage from WM microstructural alterations and underscore the importance of normal-appearing WM changes underlying cognitive and functional impairment in AD with and without cerebrovascular disease. Further developed, the combined free water and tissue neuroimaging assays could help in differential diagnosis, treatment planning, and disease monitoring of patients with mixed dementia.

**Electronic supplementary material:**

The online version of this article (doi:10.1186/s13195-017-0292-4) contains supplementary material, which is available to authorized users.

## Background

Mixed vascular and neurodegenerative dementia such as patients with Alzheimer’s disease (AD) with concomitant cerebrovascular disease (CeVD) has emerged as the leading cause of age-related cognitive impairment [1]. Accumulating evidence suggests that AD and CeVD share multiple risk factors and overlap neuropathologically, leading to additive or synergistic effects on cognitive decline [2]. White matter hyperintensity (WMH), which is associated with increased water content and vascular changes [3], serves as an important clinical diagnostic criterion by which to identify CeVD status [4]. WMH may be due to vessel disease causing infarction or a failure in the clearance of interstitial fluid from white matter (WM), which is associated with blood-brain barrier (BBB) permeability modulation [5]. However, WMH visual rating suffers from interrater variability. More importantly, WMH usually reflects a severe water increase such as edema. It is not sensitive to detection of changes, owing to small vessel damage and inflammation, particularly in normal-appearing WM [6, 7], which might exacerbate neurological dysfunction and brain damage in dementia.

It is thus important to isolate WM degeneration and vascular changes in mixed vascular and neurodegenerative dementia. Diffusion-weighted magnetic resonance imaging (MRI) has been used to examine WM microstructural alterations in dementia [8]. However, the conventional diffusion tensor imaging (DTI) indices, such as fractional anisotropy (FA) or axonal diffusivity (DA), provide inconsistent findings [7, 8], partly because changes in these indices stem from both tissue degeneration (i.e., axonal damage and demyelination) [9] and excessive extracellular fluid [10, 11]. Recently, the free water (FW) imaging method for obtaining diffusion-weighted MRI data has been proposed to correct each voxel for contamination from freely diffusing extracellular water molecules [12]. The resulting fractional volume of the FW compartment estimates the extracellular water content, and the FW-corrected DTI metrics represent microstructural tissue changes [13]. Compared with WMH, voxel-based FW increases may be more sensitive to mild vascular problems in normal-appearing tissue, including neuroinflammation and BBB permeability modulation [6].

In the context of mixed vascular and neurodegenerative dementia, it is difficult to define which neuropathological changes contribute to cognitive decline, as well as the degree to which these changes contribute to cognitive decline, because of the heterogeneous localization of lesions and the coexistence of multiple pathologies [14]. Although the FW imaging method has revealed whole-brain FW increases in AD and mild cognitive impairment (MCI) [15–17], there is a lack of understanding of the differential patterns of FW increases, especially in the normal-appearing WM, as well as microstructural changes in AD with and without CeVD. In the present study, we examined extracellular and tissue-related WM abnormalities separately in a cohort of patients with AD without CeVD (AD), AD with CeVD (AD + CeVD), and vascular dementia (VaD) using the FW imaging method. We hypothesized that patients with AD + CeVD would have higher FW values and more tissue disruption (particularly in the frontal lobe) than patients with AD because of synergistic effects. Patients with AD would have greater FW in the normal-appearing WM than age-matched healthy control subjects (HC), even though their WMH burden is comparable. Last, we assessed whether and how FW increases and tissue compartment alterations contribute to symptom severity and cognitive impairment.

## Methods

### Participants

All patients were recruited from the National University Hospital of Singapore as described in our previous work [18]. The HC group was recruited from the community. The detailed diagnostic criteria for AD, AD + CeVD, and VaD, as well as the inclusion/exclusion criteria for the HC group, are provided in Additional file 1. Psychologists evaluated each participant with extensive clinical and neuropsychological evaluation (*see* our previous work [19] and Additional file 1: Supplementary Methods).

Of the 226 eligible participants recruited, 19 did not have full T1-weighted, DTI, and fluid-attenuated inversion recovery (FLAIR) scans, and 44 did not pass neuroimaging data quality control. After age and sex matching of the remaining 163 participants, a subset of 115 subjects (41 AD without CeVD, 25 AD + CeVD, 19 VaD, and 30 HC without CeVD) was included for analyses (Table 1) (Additional file 1: Figure S7). Patients who completed cognitive assessments were included in brain-cognition association analyses (Table 1).

**Table 1:** Subjects’ clinical and demographic features

Groups	HC (*n* = 30)	AD (*n* = 41)	AD + CeVD (*n* = 25)	VaD (*n* = 19)	Overall ANOVA (*F* test)
Age, years	71.4 (6.7)	74.9 (8.0)	75.7 (5.3)	74.1 (7.2)	2.4
Sex, F/M	15/15	15/26	8/17	11/8	χ^2^ = 4.3
Handedness, L/R	3/27	0/41	0/25	0/19	–
Ethnicity, C/N	28/2	33/8	18/7	15/4	χ^2^ = 4.4
CDR-SB	0.1 (0.2)	6.2 (2.2)^a^	7.2 (2.9)^a^	6.4 (2.5)^a^	69.6^b^
MMSE (maximum score 30)	28.2 (1.3)	16.8 (5.0)^a^	15.6 (4.1)^a^	17.4 (4.6) ^a^	60.3^b^
WMH visual rating	4.3 (2.3)	5.0 (1.3)	11.2 (4.5) ^a,c^	9.2 (4.6) ^a,c^	29.3^b^
Patients with cognitive scores		AD (*n* = 33)	AD + CeVD (*n* = 20)	VaD (*n* = 16)	ANOVA (*F* test)
Age		75.6 (7.6)	75.1 (5.7)	73.8 (8.4)	0.3
Sex, F/M		12/21	7/13	10/6	χ^2^ = 3.6
Handedness L/R		0/33	0/29	0/16	–
Ethnicity, C/N		25/8	14/6	13/3	χ^2^ = 0.6
CDR-SB		6.3 (2.4)	7.3 (2.9)	6.6 (2.6)	0.9
MMSE (maximum score 30)		16.4 (5.2)	15.8 (4.5)	17.4 (4.4)	0.5
Visual construction		0.09 (1.0)	−0.08 (0.9)	−0.09 (1.1)	0.3
Visual motor		0.09 (1.1)	−0.08 (0.9)	−0.08 (1.0)	0.2
Attention		0.09 (1.1)	0.02 (0.8)	−0.21 (1.1)	0.5
Executive functioning		0.17 (1.1)	−0.18 (0.9)	−0.13 (1.0)	0.9
Language		0.09 (1.1)	−0.15 (0.9)	0 (0.9)	0.4
Verbal memory		−0.07 (1.1)	−0.22 (0.6)	0.42 (1.1)	2.0
Visuospatial memory		−0.09 (1.1)	−0.21 (0.8)	0.45 (1.1)	2.3

*Abbreviations: AD* Alzheimer’s disease, *AD + CeVD* Alzheimer’s disease with cerebrovascular disease, *ANOVA* Analysis of variance, *C/N* Chinese/non-Chinese, *CDR-SB* Clinical Dementia Rating Sum of Boxes, *HC* Healthy control subjects, *MMSE* Mini Mental State Examination, *VaD* Vascular dementia, *WMH* White matter hyperintensity

Values represent mean (SD). The WMH visual rating was measured by the age-related WM change scale score. A subset of patients with complete cognitive scores was used for brain-cognition association analyses

^a^ Group mean was different from HC on the basis of post hoc pairwise comparisons (*p* < 0.05)

^b^ Group differences at *p* < 0.05 significance level

^c^ Group mean was different from the AD group (*p* < 0.05)

### Image acquisition

Each subject underwent MRI scanning at the Clinical Imaging Research Centre, National University of Singapore (3-T MAGNETOM Trio™, A Tim® System; Siemens, Germany). High-resolution T1-weighted structural MRI scans were acquired using a magnetization-prepared rapid gradient-echo sequence (192 continuous sagittal slices, repetition time [TR]/echo time [TE]/inversion time [TI] 2300/1.9/900 milliseconds, flip angle 9 degrees, field of view [FOV] 256 × 256 mm^2^, matrix 256 × 256, isotropic voxel size 1.0 × 1.0 × 1.0 mm^3^, bandwidth 240 Hz/pixel). FLAIR images were also acquired (TR 11,000 milliseconds, TE 125 milliseconds, TI 2800 milliseconds, sensitivity encoding factor 1.5, voxel size 1.02 × 1.02 mm^2^, 60 slices, slice thickness 2.5 mm). Diffusion-weighted MRI was performed using a single-shot fast-spin echo planar imaging sequence (TR 6800 milliseconds, slices 48, thickness 3.0 mm, FOV 256 × 256 mm^2^, voxel size 3 mm^3^, *b* value 1150 seconds/mm^2^, 61 diffusion directions, 7 b0).

### DTI image processing

The DTI data were preprocessed following our previously described methods [20, 21] using the FSL tool library (http://www.fmrib.ox.ac.uk/fsl) (Additional file 1: Figure S1, step 1a). Head movements and eddy current distortions were corrected via affine registration of the DTI image to the first *b* = 0 volume. Data were discarded when the maximum displacement was greater than 3 mm. The diffusion gradients were rotated to compensate for the registration. Individual maps were visually inspected for signal dropout and artifacts. DTI index images, including FA, radial diffusivity (DR), and DA, were created by fitting the diffusion tensor model to each voxel. We subsequently used tract-based spatial statistics [22] to carry out a whole-brain voxel-wise analysis within the major WM pathways. The FA images were first nonlinearly registered to the high-resolution FMRIB58_FA image and subsequently skeletonized. The subject-level skeletonized DA and DR images were derived by projecting onto the same skeleton [21] (Additional file 1: Supplementary Methods, Figure S1, step 1a).

### WMH quantification

We performed WMH segmentation on FLAIR images using an automated procedure [23] (Additional file 1: Supplementary Methods, Figure S1, step 1c). We defined the WMH ratio as the total number of identified WMH voxels divided by the total white matter volume (WMV).

### FW imaging method

We employed the FW imaging method, which fits the eddy current-corrected DTI in each voxel to a two-compartment model (i.e., separating an FW compartment from the FW-corrected DTI tissue compartment) [12] (Additional file 1: Figure S1, step 1b). Briefly, the FW compartment models water molecules that are free to diffuse and are not restricted or hindered during the diffusion experiment. Because of the fast diffusivity and short diffusion time, FW molecules are expected only at large-enough extracellular spaces. This FW compartment has a fixed diffusivity of 3 × 10^−3^ mm^2^/second (the diffusion coefficient of the FW at body temperature). The fractional volume of this compartment in each voxel forms the FW map. The FW-corrected DTI compartment models water molecules in the proximity of the cellular membranes of brain tissues using a diffusion tensor, which is corrected for contamination by freely diffusing extracellular water, and it is thus expected to be more sensitive and more specific to axonal and myelination changes than the original DTI measures [24]. In-house MATLAB (MathWorks, Natick, MA, USA) scripts were developed for the FW analysis following our previous approaches [13, 25].

Individual voxel-wise FW and FW-corrected tissue compartment DTI maps (free water-corrected fractional anisotropy [FA_T_], free water-corrected axial diffusivity [DA_T_], and free water-corrected radial diffusivity [DR_T_]) were aligned to a standard template and projected onto the standardized FA skeleton, resulting in subject-level skeletonized images [12]. Boundary-based registration [22] was used to register individual FLAIR maps onto the DTI space. Then, both FLAIR-based WMH lesion masks and FW maps were normalized to the Montreal Neurological Institute standard space on the basis of transformations derived from FA maps. Mean FW values in the whole WM skeleton and the normal-appearing WM regions were derived (Additional file 1: Figure S1, step 3).

### Statistical analyses

#### Group comparisons of WM abnormalities

To assess group differences in WMH ratios and mean WM FW values (Additional file 1: Figure S1, step 2), we performed analysis of variance followed by post hoc pairwise group comparisons using Tukey’s test. To assess region-specific group differences in the WM indices (including FW values, original DTI indices, and FW-corrected DTI indices), following tract-based spatial statistics, we constructed voxel-wise generalized linear models (GLMs) with skeletonized WM index maps as the dependent variable; group as the independent variable; and age, sex, handedness, and ethnicity as covariates. Group differences were identified through permutation-based nonparametric testing (FSL randomise) (thresholded at *p* < 0.01, threshold-free cluster enhancement [TFCE], and family-wise error [FWE] correction [26]). Anatomical localization was determined with reference to the Johns Hopkins University WM atlas [27].

#### Associations between WM measures and dementia severity

We calculated the associations of Clinical Dementia Rating Sum of Boxes (CDR-SB) scores with mean FW values in WM or normal-appearing WM as well as DTI metrics across all patients using Pearson’s correlations (Additional file 1: Figures S1, step 4). Spearman’s correlations were used to test if WMH ratio was related to dementia severity (Additional file 1: Supplementary Methods).

#### Associations between WM measures and cognitive impairment

Pearson’s correlations were calculated between general cognitive performance (Mini Mental State Examination scores) and mean FW in WM or normal-appearing WM across all patients (Additional file 1: Figure S1, step 6). To assess region-specific WM changes underlying cognitive impairment across all patients, we built GLMs (FSL randomise) with the skeletonized FW and FA_T_ images as the dependent variables and domain-specific cognitive z-scores as the independent variables (*p* < 0.01 with TFCE and FWE correction). In all brain-behavior association analyses, age, sex, and ethnicity were included as nuisance variables (handedness was excluded because all patients were right-handed).

## Results

### Group differences in FW

**Fig. 1:**
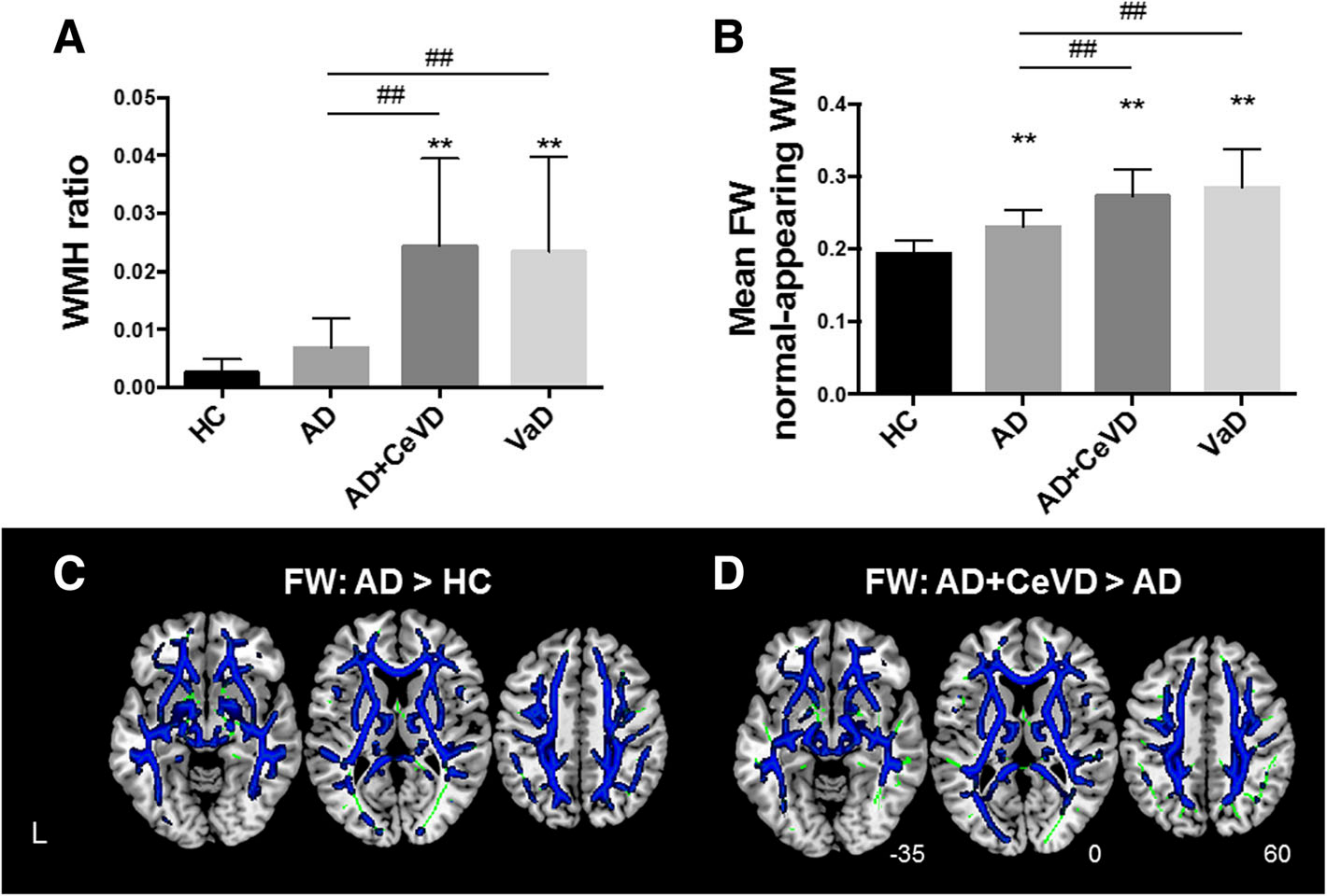
Increased free water (FW) in normal-appearing white matter (WM) regions in patients with Alzheimer’s disease (AD) compared with healthy control subjects (HC). **a** Only patients AD and cerebrovascular disease (AD + CeVD) and patients with vascular dementia (VaD) had higher white matter hyperintensity (WMH) ratios than the patients with AD (##) and HC (**), but patients with AD without CeVD did not differ from HC. **b** There was increased FW in WM in the patients with AD with and without CeVD and in the patients with VaD compared with the HC (**). The CeVD groups had further FW increases compared with the patients with AD. All data are reported at p < 0.01 corrected. **c** The patients with AD had widespread FW increases in normal-appearing WM regions compared with the HC, reflecting mild vascular changes (*blue*). **d** The patients with AD + CeVD had higher FW than the patients with AD (*blue*; p < 0.01 threshold-free cluster enhancement- and family-wise error-corrected). The WM skeleton is highlighted in *green*

WMH autosegmentation confirmed that the patients with AD + CeVD and patients with VaD had greater WMH ratios than the patients with AD and the HC (Fig. 1a). Consistent with the WMH ratio, the patients with AD + CeVD and patients with VaD had greater mean WM FW than the patients with AD and HC (Additional file 1: Figure S2), which was further confirmed by whole-brain voxel-wise comparisons (Fig. 1d and Additional file 1: Figure S3, Table S1). As expected, the patients with AD had higher FW than the HC in most WM regions, except in the occipital fibers (Fig. 1c and Additional file 1: Figure S2, Table S1), in contrast to the similar WMH ratios between the patients with AD and HC.

More important, patients with AD + CeVD and patients with VaD had greater mean FW in the normal-appearing WM than patients with AD and HC. Patients with AD had higher FW in the normal-appearing WM than HC (Fig. 1b). Patients with AD + CeVD and patients with VaD did not differ in their WMH ratios or FW values. To minimize the concern that FW can be affected by brain volume changes, we repeated the analyses, controlling for the total WMV. Most of the group differences remained (Additional file 1: Table S1).

### FW increases correlated with symptom severity

**Fig. 2:**
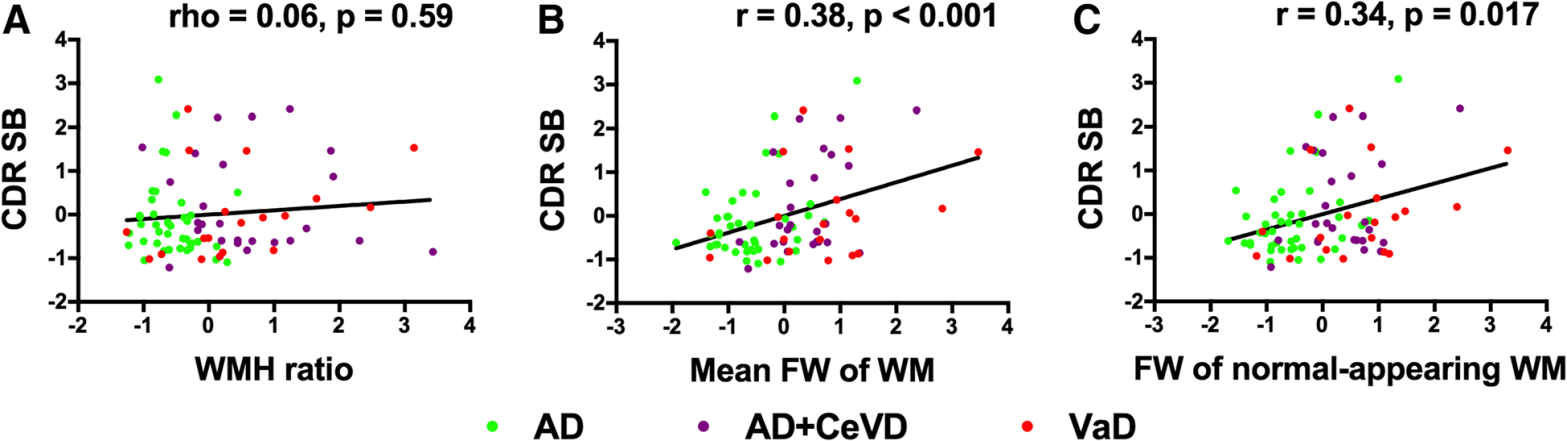
Free water (FW) increases are associated with dementia severity. **a** There was no association between white matter hyperintensity (WMH) and Clinical Dementia Rating Sum of Boxes (CDR-SB) score. **b** Increased FW values across all white matter (WM) regions was related to increased CDR-SB scores across all patients. **c** Increased FW values in the normal-appearing WM were related to increased CDR-SB scores across all patients. *CeVD* Cerebrovascular disease, *VaD* Vascular dementia

We identified a strong correlation between the mean FW values (averaged across WM skeleton) and dementia severity across all patients; that is, increased FW was related to higher CDR-SB scores (*r* = 0.38, *p* < 0.001) (Fig. 2b). The association remained after controlling for WMV (*r* = 0.34, *p* = 0.012). Similar associations were found within the AD (*r* = 0.41, *p* = 0.008) and AD + CeVD (*r* = 0.47, *p* = 0.017) groups, but not for the VaD group (*r* = 0.31, *p* = 0.18).

Further, the increased FW in normal-appearing WM was related to higher CDR-SB scores across all patients (*r* = 0.34, *p* = 0.017) (Fig. 2c). The association remained after controlling for WMV (*r* = 0.29, *p* = 0.018). The associations were found within the AD group (*r* = 0.39, *p* = 0.011), but not for the AD + CeVD (*r* = 0.26, *p* = 0.21) or VaD groups (*r* = 0.34, *p* = 0.16). In contrast, the WMH ratio was not associated with dementia severity (Fig. 2a).

### Abnormalities in original and FW-corrected DTI metrics

**Fig. 3:**
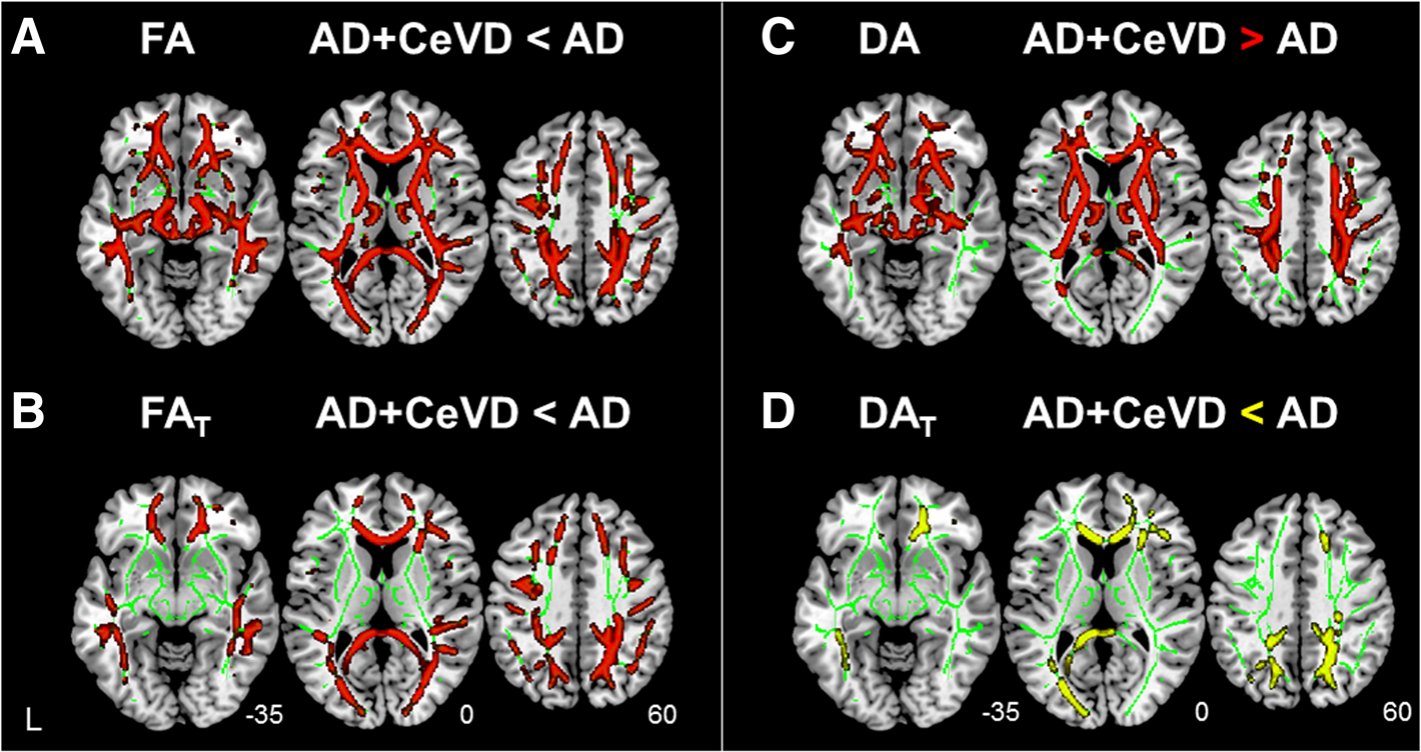
Free water (FW)-corrected measures revealed more lobar white matter (WM) tissue-related microstructural damage in patients with Alzheimer’s disease with cerebrovascular disease (AD + CeVD) than in patients with AD without CeVD. **a** On the basis of the conventional diffusion tensor imaging (DTI) model, patients with AD + CeVD exhibited reduced fractional anisotropy (FA) (*red*) compared with patients with AD in most of the WM regions, including the lobar and subcortical fibers. **b** Free water-corrected fractional anisotropy (FAT) revealed more lobar WM microstructural damage in patients with AD + CeVD than in patients with AD without CeVD, sparing the subcortical and brainstem regions. The WM skeleton is highlighted in green. **c** On the basis of the original DTI model, the patients with AD + CeVD had widespread increased axial diffusivity (DA) compared with patients with AD (*red*). **d** Following FW correction, a focal lobar (frontal and occipital lobes) free water-corrected axial diffusivity (DAT) reduction was identified in patients with AD + CeVD compared with patients with AD (*yellow*). The white matter skeleton is highlighted in *green*. Results are reported at p < 0.01, threshold-free cluster enhancement- and family-wise error-corrected

On the basis of conventional DTI metrics, patients with AD + CeVD and patients with VaD had widespread WM FA reductions in both lobar and subcortical regions compared with patients with AD (Fig. 3a and Additional file 1: Figure S4A and Table S2). Nevertheless, the FW-corrected tissue compartment analyses showed that patients with AD + CeVD and patients with VaD had only lobar FA_T_ reductions, in contrast to patients with AD (less extensive than the original metrics), sparing the subcortical and brainstem regions (Fig. 3b and Additional file 1: Figure S4B and Table S2). There was no difference in FA or FA_T_ between the AD + CeVD and VaD groups.

Compared with HC, all three dementia subtypes had reduced FA (Additional file 1: Figure S5A, *red*, and Table S2). None of the areas had increased FA in patients compared with HC. A further analysis of the FW-corrected tissue compartments showed that the FA_T_ changes in the three dementia subgroups became less extensive than the original DTI results compared with the HC. Specifically, several subcortical and brainstem fibers no longer exhibited WM microstructural damage in patients compared with HC (Additional file 1: Figure S5A, *yellow*, and Table S2).

Interestingly, on the basis of conventional DTI metrics, patients with AD + CeVD and patients with VaD had higher DA values than patients with AD, particularly in the frontal-subcortical and brainstem regions (Fig. 3c and Additional file 1: Figure S4C and Table S3). In contrast, after FW correction, patients with AD + CeVD and patients with VaD had lower DA_T_ values than patients with AD in the frontal and occipital regions; the subcortical and brainstem fibers no longer exhibited any DA_T_ group differences (Fig. 3d and Additional file 1: Figure S4D and Table S3). Again, there was no difference in DA/DA_T_ between the AD + CeVD and VaD groups.

All three dementia subtypes had increased DA compared with HC in the temporal and subcortical regions and parts of the frontal and parietal lobes (Additional file 1: Figure S5B, *red*, and Table S3). None of the areas had reduced DA in patients compared with HC. In contrast, after FW correction, DA_T_ in the frontal, parietal, and occipital lobes was decreased for all three patient groups compared with HC (Additional file 1: Figure S5B, *green*, and Table S3). None of the areas had increased DA_T_ in the patients. The differences between the DR and DR_T_ patterns are described in Additional file 1: Supplementary Results.

### Correlations between WM abnormalities and cognitive performance

**Fig. 4:**
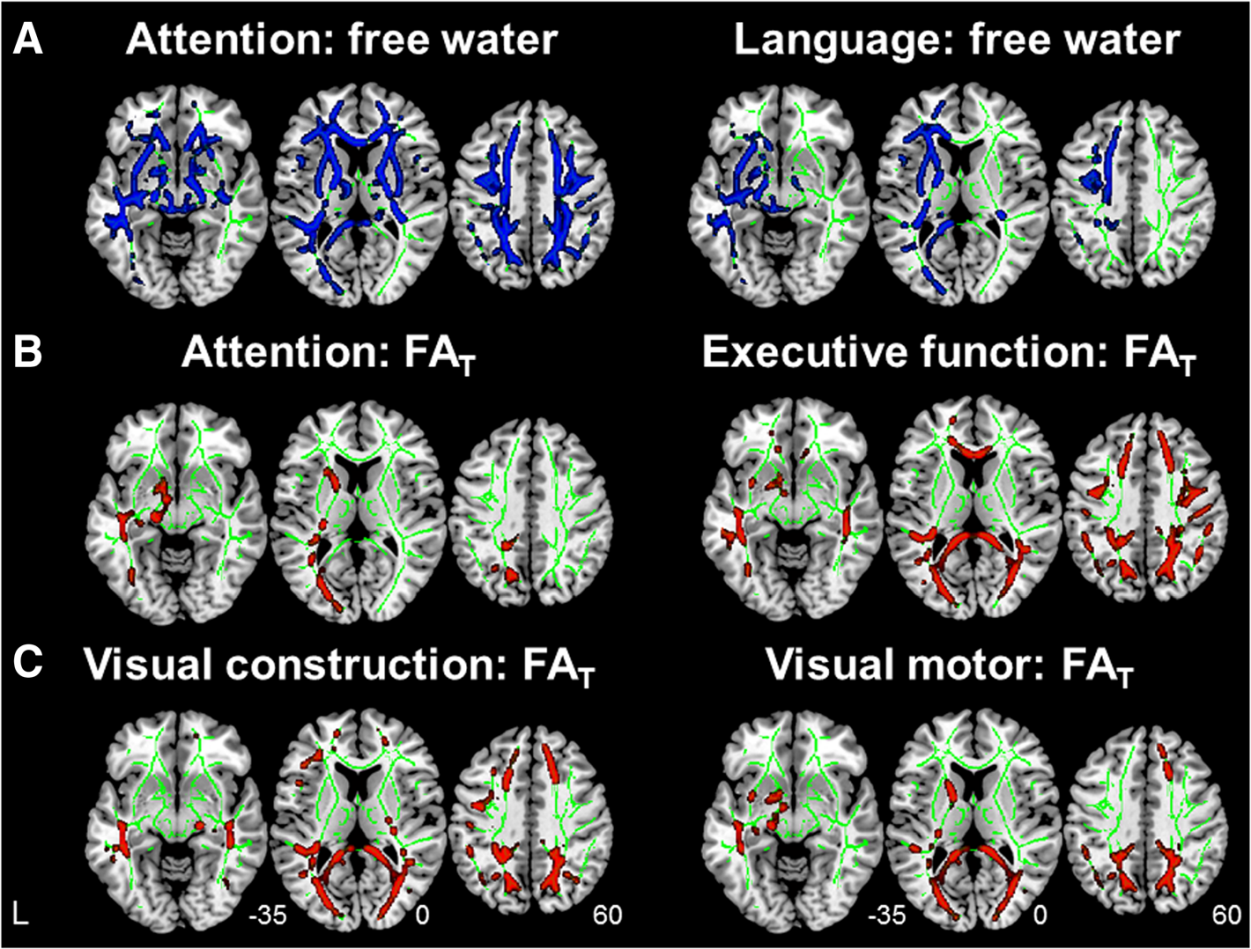
Free water (FW) increases and FW-corrected tissue compartment deterioration correlated with cognitive deficits. **a** Whole-brain voxel-wise linear regression indicated that increased FW values in widespread brain white matter regions were associated with worse cognitive performance, including attention, executive functioning, visual construction, and visuomotor performance (*blue*; attention as an example; see also Additional file 1: Table S5). Only FW increase in the left hemisphere was associated with language deficits. Moreover, region-specific FW-corrected tissue compartment damage was associated with deficits in attention, executive functioning (**b**), visual construction, and visuomotor (**c**) domains. Results are reported at p < 0.01 significance level, threshold-free cluster enhancement- and family-wise error-corrected. *FA*_*T*_ Free water-corrected fractional anisotropy

The whole-brain voxel-wise GLM analyses revealed that higher FW values in nearly all WM regions were associated with poorer executive functioning, visual construction, and visuomotor performance (Additional file 1: Table S5). Attention was negatively correlated with the FW values in most WM fibers, except the right occipital and temporal regions, whereas lower language performance was associated with higher FW values in the left hemisphere (Fig. 4a). Similar findings were observed after controlling for WMV (Additional file 1: Table S5).

In contrast to the widespread patterns in FW-cognition associations, the FW-corrected tissue compartment had region-selective associations with cognitive performance. Specifically, attention decline was associated with reduced FA_T_ in the left hemisphere only (including the middle parietal, occipital, callosal, and subcortical fibers) (Fig. 4b and Additional file 1: Table S5). Executive dysfunction was related to lower FA_T_ in the bilateral frontal, parietal, and occipital fibers (Fig. 4b and Additional file 1: Table S5). Visual construction impairment was related to lower FA_T_ in the frontal, parietal, and occipital regions, whereas visuomotor deficits were related to lower FA_T_ mostly in the occipital and subcortical regions (Fig. 4c and Additional file 1: Table S5).

Interestingly, increased FW in brainstem fibers was associated with all domains of cognitive dysfunction, but decreased FA_T_ in the brainstem regions was not. There were no associations of FW/FA_T_ with memory performance due to the flooring effect, because most patients with dementia had severe memory deficits. The associations between the FW and DTI metrics and the general cognitive measures are described in Additional file 1: Supplementary Results.

## Discussion

Our findings provide new insights into differential WM microstructural and FW alterations in normal-appearing tissue in mixed vascular and neurodegenerative dementia. All patients, including patients with AD without CeVD, had increased FW compared with HC. Importantly, increased FW was detected in normal-appearing WM regions in patients with AD, reflecting mild vascular changes. Widespread FW increases in the whole WM and normal-appearing WM (but not the WMH ratio) were associated with symptom severity. Both lobar and subcortical FW increases were associated with poorer cognitive performance, such as in attention and executive functioning, whereas only left hemispheric FW increases were related to language deficits. In parallel, compared with the original DTI metrics, which might overestimate axonal damage or demyelination, the FW-corrected tissue compartment had better clinical specificity because it showed tissue-specific microstructural differences between the CeVD and non-CeVD groups. In addition, these region-specific tissue changes were associated with poorer cognitive performance. Our findings underscore the value of FW corrections in isolating vascular damage from WM microstructural alterations and help connect both extracellular FW and microstructural abnormalities with cognitive impairment in mixed vascular and neurodegenerative dementia.

As far as we know, this is the first study demonstrating that the FW measure is sensitive to vascular changes because it successfully distinguished the non-CeVD from the CeVD groups, corresponding well with their WMH ratio differences. Moreover, we further demonstrate that patients with AD had widespread FW increases compared with HC, particularly in the normal-appearing WM. Previous studies showed similar FW increases in AD [15], but they did not reveal whether the FW increase is due to WM lesions or to changes in the normal-appearing WM. Our findings of increased FW in normal-appearing WM in the AD without CeVD group suggest mild vascular damage that may be due to microvascular degeneration [28] and neuroinflammation-related BBB permeability modulation [29] in normal-looking WM tissue in AD. Both imaging and postmortem studies have indicated that vascular pathology exists in AD [2, 29]. Deposition of amyloid-β (Aβ) might lead to vascular problems such as BBB breakdown and neurovascular regulation impairment. The pathogenic chain of these vascular effects, in a vicious cycle, produces further Aβ deposition and neurofibrillary tangle formation. Together, these pathological factors may contribute to the later development of neurodegeneration and cognitive decline in AD [30]. Other possible causes of FW increases might include abnormally low cell density, low dendrite number and volume, and WM degeneration [25, 31–34]. However, group differences in FW remained after controlling for the total WMV.

Critically, the mean FW value, both across the whole WM and in the normal-appearing WM, was associated with symptom severity, whereas the WMH ratio was not. Although both WMH and FW reflect accumulating water within the WM [5, 12], WMH reflects only severe water increases such as edema [6]. Mild increases in FLAIR image contrast may be hard to identify. Further, the segmented WMH is often surrounded by a penumbra of subtly injured tissue with mild water content increases. These changes cannot be accurately quantified by the WMH volume [35]. In contrast, our findings suggest that the FW metric can detect these mild water content increases in a voxel-based manner, which might contribute to the etiology of AD [36]. Therefore, the FW compartment provides a more sensitive way of detecting mild vascular changes in normal-appearing WM regions in dementia, which could potentially help in differential diagnosis and neuropathological understanding of mixed vascular and neurodegenerative dementia.

The second important finding is that the FW-corrected DTI metrics revealed region-specific WM microstructural differences between the CeVD and non-CeVD dementia groups. CeVD-related axonal damage and demyelination have been reported in postmortem studies [37]. However, recent data suggest that DTI-based metrics may not correspond well with the axonal pathology measured in patients with CeVD [38]. This is in line with our observations that the original DTI metrics identified nonspecific and global WM microstructural differences between the CeVD and non-CeVD groups. Increases in the FW compartment could mask the true alterations in WM fibers [16] (i.e., loss of oligodendrocytes, myelin sheaths, axons, and neurons) and could even overestimate tissue damage, particularly in the subcortical and brainstem regions [24]. Therefore, on the basis of FW-corrected DTI metrics, the patients with AD + CeVD had more focal lobar damage (FA_T_ decrease and DR_T_ increase) in the occipital and frontal lobes than the patients with AD. Moreover, in previous DTI studies, researchers reported inconsistent DA changes (both increases and decreases) in patients with AD and patients with MCI compared with control subjects [8, 9]. This inconsistency is partly due to the fact that increased extracellular fluid may lead to increased DA [10, 11]. Using the FW correction method, we confirmed lower DA_T_ in patients with dementia than in HC as well as lower DA_T_ in the frontal and occipital regions in the CeVD group than in the non-CeVD group. This evidence lends further support to the accumulating evidence that CeVD exacerbates neurological dysfunction and brain damage in AD [30].

The next question is whether and how FW and tissue compartment changes contribute to cognitive impairment. A recent study illustrated that FW was associated with cognitive performance in Parkinson’s disease [39]. Only two studies showed associations between tissue compartment changes and cognition in predefined regions in MCI: Tissue fraction in the parahippocampal cingulum was associated with memory decline [17], and tissue compartment measures in the anterior cingulum were associated with cognitive control [40]. We found a region-specific influence of extracellular and microstructural changes on cognitive decline across patients with AD with and without CeVD using a whole-brain search. Widespread FW increases (both lobar and subcortical/brainstem) appear to be related to poor cognitive performance, particularly executive functioning, attention, visuomotor performance, and visual construction. Moreover, consistent with the literature regarding a dominant role of the left hemisphere in language [41], only left hemispheric FW increases (but not tissue damage) were related to language deficits. In contrast, only focal lobar FW-corrected WM microstructural damage was associated with each cognitive domain, suggesting distinct default mode and executive control network disruptions in non-CeVD [42, 43] and CeVD groups [44]. Such differential associations of vascular and WM microstructure with cognition reveal the possible neural mechanism in patients with mixed vascular and neural degeneration.

Some limitations of the study should be noted. The possible influence of WM degeneration on FW increases could not be completely excluded [34]. However, to mitigate this concern, we controlled for total WMV in the FW analyses, and the results remained. In addition, we did not examine amyloid deposition in our patients. Diagnoses of AD and VaD based on conventional clinical criteria may lead to heterogeneous groups with overlapping pathologies.

## Conclusions

We have demonstrated the importance of FW analysis in separating vascular-related extracellular FW increases and WM microstructural disruptions in mixed vascular and neurodegenerative dementia. Importantly, these FW increases in normal-appearing WM contribute to dementia severity and cognitive deficits. Future longitudinal studies on preclinical AD with and without CeVD are needed to examine the early changes, temporal trajectories, and possible interplay between vascular and tissue abnormalities. Further developed, the combined FW and tissue assays could help in the differential diagnosis, individual treatment planning, and disease monitoring for patients with mixed dementia.

## Additional file

Additional file 1: Supplementary methods, results, tables, and figures. (DOCX 4194 kb)

## Abbreviations

Aβ: Amyloid-β
AD: Alzheimer’s disease
AD + CeVD: Alzheimer’s disease with cerebrovascular disease
ANOVA: Analysis of variance
BBB: Blood-brain barrier
CDR-SB: Clinical Dementia Rating Sum of Boxes
CeVD: Cerebrovascular disease
C/N: Chinese/non-Chinese
DA: Axial diffusivity
DA_T_: Free water-corrected axial diffusivity
DR: Radial diffusivity
DR_T_: Free water-corrected radial diffusivity
DTI: Diffusion tensor imaging
FA: Fractional anisotropy
FA_T_: Free water-corrected fractional anisotropy
FLAIR: Fluid-attenuated inversion recovery
FOV: Field of view
FW: Free water
FWE: Family-wise error
GLM: Generalized linear model
HC: Healthy control subjects
MCI: Mild cognitive impairment
MMSE: Mini Mental State Examination
MRI: Magnetic resonance imaging
TE: Echo time
TFCE: Threshold-free cluster enhancement
TI: Inversion time
TR: Repetition time
VaD: Vascular dementia
WM: White matter
WMH: White matter hyperintensity
WMV: White matter volume
