# Supplementary material for: Distinct white matter microstructural abnormalities and extracellular water increases relate to cognitive impairment in Alzheimer’s disease with and without cerebrovascular disease
Source: Alzheimers Res Ther. 2017 Aug 17;9:63. doi: 10.1186/s13195-017-0292-4 (PMC5561637; doi:10.1186/s13195-017-0292-4)
Supplement: Additional file 1: — Supplementary methods, results, tables, and figures. (DOCX 4194 kb) [file 13195_2017_292_MOESM1_ESM.docx]

**SUPPLEMENTARY MATERIALS**

**Supplementary Methods**

**1. Diagnoses and inclusion/exclusion criteria**

Each participant underwent extensive clinical and neuropsychological evaluation including the Clinical Dementia Rating Scale (CDR), the Mini-Mental State Examination (MMSE), the Montreal Cognitive Assessment, the informant questionnaire on cognitive decline and a formal neuropsychological battery, which had been locally validated for older Singaporeans.

Diagnoses of dementia were made at weekly consensus meetings, following a review of the patients’ clinical history, blood work, neuropsychological assessments and neuroimaging data, which were conducted by neurologists, neuropsychologists, research nurses, and research assistants following our previous work[1]. Diagnoses were consistent with the Diagnostic and Statistical Manual of Mental Disorders (DSM) -IV criteria. Computed tomography (CT), magnetic resonance imaging (MRI), and magnetic resonance angiography were reviewed as part of the diagnostic process. Etiological diagnoses were made according to the National Institute of Neurological and Communicative Disorders and Stroke and the Alzheimer’s disease (AD) and Related Disorders Association guidelines for AD. Briefly, AD patients were classified based on a gradual and slow onset of memory problems, impairment in objective neuropsychological assessment, and loss in activities of daily living. The National Institute of Neurological Disorders and Stroke–Association Internationale pour la Recherche en l’Enseignement en Neurosciences criteria were used for vascular dementia (VaD). According to these criteria, VaD was diagnosed based on a history of stroke temporally related to the onset or worsening of cognitive impairment, neuroimaging evidence of extensive or multiple infarcts, and loss in activities of daily living[1]. Neuroimaging evidence for significant cerebrovascular disease was assessed using the following criteria: (1) cortical infarcts; (2) two or more lacunes; and (3) confluent white matter (WM) lesions in two brain regions (Age Related WM Change scale score ≥ 8)[2]. For the healthy controls (HC), we ensured that the participants had no impairment in the seven domains assessed via extensive neuropsychological tests, and their Mini-Mental State Examination (MMSE) scores were greater than or equal to 26[3, 4].

Participants were excluded from this study if they were/had: (1) hypoxic/ anoxic, hypotensive, hypertensive, uremic or hepatic encephalopathy; (2) traumatic, nutritional or toxic disorders that affect the central nervous system (CNS); (3) current substance use disorders (e.g., alcohol, barbiturates, opiates, amphetamines, phencyclidine, and/or cocaine as defined by the DSM-IV) or previous substance use disorders that affect the CNS; (4) intracerebral hemorrhages that may cause cognitive impairments; (5) cranial arteritis, CNS inflammatory vasculitides, or Moyamoya disease; (6) CNS infections, including syphilis, tuberculosis, fungi, rickettsiae, bacterium, viral encephalitis, Creutzfeld-Jacob disease or other CNS infections; (7) space-occupying intracranial mass lesions; (8) obstructive or normal pressure hydrocephalus; (9) difficulty controlling epilepsy, which may cause cognitive impairments; or (10) schizophrenia or bipolar disorder.

**2. White matter hyperintensity (WMH) quantification**

The WMH quantification steps included: 1) register the FLAIR image to T1-weighted structural MRI for each individual via linear transformation based on normalized mutual information; 2) segment grey matter (GM), WM, and cerebral spinal fluid (CSF) from the T1-weighted structural MR images using the Statistical Parametric Mapping (SPM8) software (<http://www.fil.ion.ucl.ac.uk/spm/)>; 3) apply this segmentation on the FLAIR image and remove the non-brain regions; 4) determine the modal pixel intensity within the masked FLAIR image; and 5) perform a threshold-based segmentation by identifying all voxels 1.45 times or larger than the modal pixel intensity. The total white matter volume per subject was also derived.

**3. Associations between the diffusion measures and symptom severity**

The mean FA, FA_T_, DA, DA_T_, DR, and DR_T_ were derived from the WM skeletonized regions showing group differences in a one-way ANOVA analysis (Randomise, thresholded at p < 0.01 with a threshold-free cluster enhancement (TFCE) and a family-wise error (FWE) correction). Pearson’s correlations were calculated between the symptom severity (CDR sum-of-boxes scores) and mean DTI measures (original and FW-corrected) across all of the patients, with age, gender, and ethnicity as nuisance variables.

Furthermore, to test whether FW increases could partially explain some variance in the standard DTI metrics (e.g., FA) and contribute to the associations between standard DTI measures and cognitive measures, we constructed the linear regression models across all patients with the CDR-SB (or MMSE) as the dependent variable, the FW and standard DTI indices (FA, DR or DA in separate models) as the independent variables, and age, gender, and ethnicity as covariates.

**Supplementary Results**

**1. Demographic, clinical, and cognitive characteristics**

We analyzed the demographic, clinical, and cognitive measures using SPSS. The results are reported at a significance level of *p<*0.05. There were no differences in age, gender, ethnicity and handedness across the four groups (Table 1). As expected, the MMSE scores were lower and the CDR-SB scores were higher in the dementia patients compared with HC. There were no differences in the MMSE scores, CDR-SB scores, and domain-specific cognitive z-scores between the three dementia groups.

**2. Abnormalities in DTI and FW-corrected DR in dementia subtypes**

Based on the original DTI metrics, all three dementia subtypes had increased DR compared to the HC (Supplementary Fig. 6A, red color, Supplementary Table 4). The AD+CeVD and VaD patients had more widespread DR increases than the AD patients (Supplementary Fig. 6B, red color, Supplementary Table 4). No areas had reduced DR or DR_T_ in the patients. After the FW correction, the DR_T_ changes in the three dementia subgroups compared to the HC became less extensive than the original DTI results (Supplementary Fig. 6A, yellow color, Supplementary Table 4). Similarly, the AD+CeVD and VaD patients had more widespread DR_T_ increases than the AD patients, but the differences were less significant than the DTI findings (Supplementary Fig. VIB, yellow color, Supplementary Table IV).

There was no difference between the AD+CeVD and VaD groups in any of the measures.

**3. Associations of white matter FW and DTI metrics with the clinical measures**

Increased FW and reduced FA_T_ (derived from all regions showing group differences) were associated with lower MMSE score across all patients (FW in WM: r=-0.27, p=0.013; FW in the normal-appearing WM: r=-0.26, p=0.015; FA_T_: r=0.25, p=0.025).

The mean WM DTI metrics (derived from the clusters with group differences) were correlated with the CDR sum-of-boxes scores across all of the patients, but the FW-corrected diffusion metrics were not (Supplementary Table 6).

The mean WM DTI metrics and FW-corrected diffusion metrics (derived from the clusters with group differences), except for DA_T_, were correlated with the MMSE scores across all of the patients (Supplementary Table 6).

In addition, we found that, when putting in the same model, FW increases but not the standard DTI indices were associated with CDR-SB (or MMSE) (Supplementary Table 7). These findings suggest that the associations between the standard DTI measures and clinical measures might be largely driven by the FW increases. Thus, after FW correction, the FW-corrected DTI measures (reflecting the actual tissue microstructural changes) had weakened relationship with clinical measures (Supplementary Table 6).

**Table S1. Regions of abnormal free-water (FW) compartments in the dementia patients.** The WM clusters in which the FW or FW controlled for white matter volume (denoted by FWw) exhibited group differences (TFCE and FWE corrected, p < 0.01, with a minimum cluster size of 10 voxels) are listed with the MNI coordinates (mm) and t-statistics of the peak voxel. **Abbreviations:** left hemisphere = L, right hemisphere = R, corticospinal tract = CST, cerebral peduncle R = CP, anterior limb of internal capsule = ALIC, posterior limb of internal capsule = PLIC, retrolenticular part of internal capsule = RLIC, anterior corona radiata = ACR, superior corona radiata = SCR, posterior corona radiata = PCR, posterior thalamic radiation (include optic radiation) = PTR, external capsule = EC, sagittal stratum (includes inferior longitudinal fasciculus and inferior fronto-occipital fasciculus) = SS, superior longitudinal fasciculus = SLF, superior fronto-occipital fasciculus (may be part of the anterior internal capsule) = SFO, uncinate fasciculus = UF, fornix (column and body of the fornix) = Fx, cingulum (cingulate gyrus) = CCG, cingulum (hippocampus) = CHIP, fornix (cres)/stria terminalis (cannot be resolved with the current resolution) = Fx/ST, genu of the corpus callosum = gCC, body of the corpus callosum = bCC, splenium of the corpus callosum = sCC, tapetum = TAP, middle cerebellar peduncle = MCP, pontine crossing tract (a part of the MCP) = PCT, medial lemniscus = ML, inferior cerebellar peduncle = ICP, superior cerebellar peduncle = SCP.

**Table S2. Regions of abnormal fractional anisotropy (FA) or FW-corrected FA (FA_T_) in the dementia patients.** The WM clusters in which FA or FA_T_ exhibited group differences (TFCE and FWE corrected, p < 0. 01, with a minimum cluster size of 10 voxels) are listed with the t-statistics for the peak voxel. **Abbreviations:** left hemisphere = L, right hemisphere = R, corticospinal tract = CST, cerebral peduncle R = CP, anterior limb of internal capsule = ALIC, posterior limb of internal capsule = PLIC, retrolenticular part of internal capsule = RLIC, anterior corona radiata = ACR, superior corona radiata = SCR, posterior corona radiata = PCR, posterior thalamic radiation (include optic radiation) = PTR, external capsule = EC, sagittal stratum (includes inferior longitudinal fasciculus and inferior fronto-occipital fasciculus) = SS, superior longitudinal fasciculus = SLF, superior fronto-occipital fasciculus (may be part of the anterior internal capsule) = SFO, uncinate fasciculus = UF, fornix (column and body of the fornix) = Fx, cingulum (cingulate gyrus) = CCG, cingulum (hippocampus) = CHIP, fornix (cres)/stria terminalis (cannot be resolved with the current resolution) = Fx/ST, genu of the corpus callosum = gCC, body of the corpus callosum = bCC, splenium of the corpus callosum = sCC, tapetum = TAP, middle cerebellar peduncle = MCP, pontine crossing tract (a part of the MCP) = PCT, medial lemniscus = ML, inferior cerebellar peduncle = ICP, superior cerebellar peduncle = SCP.

**Table S3. Regions of abnormal axial diffusivity (DA) or FW-corrected DA (DA_T_) in the dementia patients.** The WM clusters in which DA or DA_T_ exhibited group differences (TFCE and FWE corrected, p < 0.01, with a minimum cluster size of 10 voxels) are listed with the t-statistics for the peak voxel. Abbreviations: left hemisphere = L, right hemisphere = R, corticospinal tract = CST, cerebral peduncle R = CP, anterior limb of internal capsule = ALIC, posterior limb of internal capsule = PLIC, retrolenticular part of internal capsule = RLIC, anterior corona radiata = ACR, superior corona radiata = SCR, posterior corona radiata = PCR, posterior thalamic radiation (include optic radiation) = PTR, external capsule = EC, sagittal stratum (includes inferior longitudinal fasciculus and inferior fronto-occipital fasciculus) = SS, superior longitudinal fasciculus = SLF, superior fronto-occipital fasciculus (may be part of the anterior internal capsule) = SFO, uncinate fasciculus = UF, fornix (column and body of the fornix) = Fx, cingulum (cingulate gyrus) = CCG, cingulum (hippocampus) = CHIP, fornix (cres)/stria terminalis (cannot be resolved with the current resolution) = Fx/ST, genu of the corpus callosum = gCC, body of the corpus callosum = bCC, splenium of the corpus callosum = sCC, tapetum = TAP, middle cerebellar peduncle = MCP, pontine crossing tract (a part of the MCP) = PCT, medial lemniscus = ML, inferior cerebellar peduncle = ICP, superior cerebellar peduncle = SCP.

**Table S4. Regions of abnormal radial diffusivity (DR) or FW-corrected DR (DR_T_) in the dementia patients.** The WM clusters in which DR or DR_T_ exhibited group differences (TFCE and FWE corrected, p < 0.01, with a minimum cluster size of 10 voxels) are listed with the t-statistics for the peak voxel. **Abbreviations:** left hemisphere = L, right hemisphere = R, corticospinal tract = CST, cerebral peduncle R = CP, anterior limb of internal capsule = ALIC, posterior limb of internal capsule = PLIC, retrolenticular part of internal capsule = RLIC, anterior corona radiata = ACR, superior corona radiata = SCR, posterior corona radiata = PCR, posterior thalamic radiation (include optic radiation) = PTR, external capsule = EC, sagittal stratum (includes inferior longitudinal fasciculus and inferior fronto-occipital fasciculus) = SS, superior longitudinal fasciculus = SLF, superior fronto-occipital fasciculus (may be part of the anterior internal capsule) = SFO, uncinate fasciculus = UF, fornix (column and body of the fornix) = Fx, cingulum (cingulate gyrus) = CCG, cingulum (hippocampus) = CHIP, fornix (cres)/stria terminalis (cannot be resolved with the current resolution) = Fx/ST, genu of the corpus callosum = gCC, body of the corpus callosum = bCC, splenium of the corpus callosum = sCC, tapetum = TAP, middle cerebellar peduncle = MCP, pontine crossing tract (a part of the MCP) = PCT, medial lemniscus = ML, inferior cerebellar peduncle = ICP, superior cerebellar peduncle = SCP.

**Table S5. Regions in which the free-water (FW) and FW-corrected fractional anisotropy (FA_T_) were correlated with the cognitive scores.** The WM clusters in which the FW, FW controlling for white matter volume (FWw) and FA_T_ were positively correlated with the cognitive scores (TFCE and FWE corrected, p < 0.01, with a minimum cluster size of 10 voxels) are listed with the t-statistics for the peak voxel. **Abbreviations:** left hemisphere = L, right hemisphere = R, corticospinal tract = CST, cerebral peduncle R = CP, anterior limb of internal capsule = ALIC, posterior limb of internal capsule = PLIC, retrolenticular part of internal capsule = RLIC, anterior corona radiata = ACR, superior corona radiata = SCR, posterior corona radiata = PCR, posterior thalamic radiation (include optic radiation) = PTR, external capsule = EC, sagittal stratum (includes inferior longitudinal fasciculus and inferior fronto-occipital fasciculus) = SS, superior longitudinal fasciculus = SLF, superior fronto-occipital fasciculus (may be part of the anterior internal capsule) = SFO, uncinate fasciculus = UF, fornix (column and body of the fornix) = Fx, cingulum (cingulate gyrus) = CCG, cingulum (hippocampus) = CHIP, fornix (cres)/stria terminalis (cannot be resolved with the current resolution) = Fx/ST, genu of the corpus callosum = gCC, body of the corpus callosum = bCC, splenium of the corpus callosum = sCC, tapetum = TAP, middle cerebellar peduncle = MCP, pontine crossing tract (a part of the MCP) = PCT, medial lemniscus = ML, inferior cerebellar peduncle = ICP, superior cerebellar peduncle = SCP

**Table S6. Correlations between the mean WM diffusion metrics and the clinical measures.** Correlation of the Clinical Dementia Rating Scale sum-of-boxes (CDR-SB) scores and Mini-Mental State Examination (MMSE) scores with the diffusion and FW corrected diffusion metrics (derived from the regions showing group differences) across all of the patients. The r-values and p-values are presented in each cell and ‘*’ represents significant correlations. **Abbreviations:** FA: fractional anisotropy; DA: axial diffusivity; FA_T_: FW-corrected FA; DA_T_: FW-corrected DA.

|  | FA | DA | DR | FA_T_ | DA_T_ | DR_T_ |
| --- | --- | --- | --- | --- | --- | --- |
| CDR-SB | -0.24 | 0.25 | 0.26 | -0.22 | -0.21 | 0.21 |
|  | 0.033* | 0.026* | 0.021* | 0.053 | 0.055 | 0.054 |
| MMSE | 0.30 | -0.28 | -0.30 | 0.25 | 0.17 | -0.26 |
|  | 0.007* | 0.012* | 0.008* | 0.025* | 0.123 | 0.017* |

**Table S7. Linear regression results of free-water (FW) and the standard DTI measures with clinical measures.** The standardized beta-values (top row) and p-values (bottom row) are presented in each cell. ‘*’ represents the significance level of p< 0.05. **Abbreviations:** FA: fractional anisotropy; DA: axial diffusivity.

|  | FA | FW | DA | FW | DR | FW |
| --- | --- | --- | --- | --- | --- | --- |
| CDR-SB | 0.32 | 0.61 | -0.28 | 0.58 | -0.50 | 0.81 |
|  | 0.24 | **0.03*** | 0.35 | **0.05*** | 0.22 | **0.04*** |
| MMSE | -0.19 | -0.52 | 0.28 | -0.60 | 0.55 | -0.88 |
|  | 0.45 | **0.04*** | 0.32 | **0.04*** | 0.06 | **0.04*** |

**
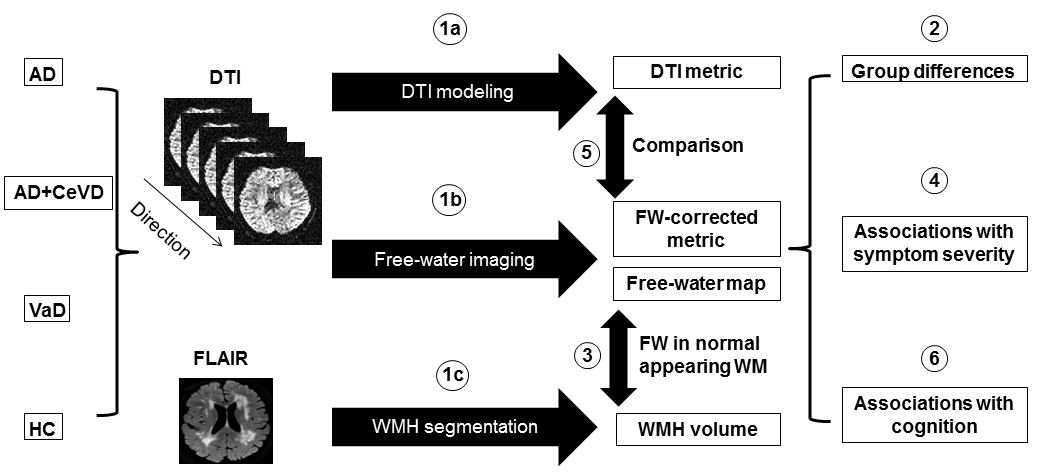
**

**Figure S1. Study design schematic.** The study included six steps: deriving (1a) the original DTI indices, (1b) FW compartment and FW-corrected DTI indices, and (1c) WMH ratio for each participant; (2) performing the whole-brain voxel-wise group comparisons on the original and FW-corrected DTI metrics between the three dementia groups and HC; (3) deriving the mean FW values in the normal-appearing WM from each group; (4) correlating the FW, FW-corrected DTI indices and WMH ratios with symptom severity in patients; (5) comparing the DTI indices with the FW-corrected DTI indices; and (6) correlating the FW and FW-corrected DTI indices with cognitive performance. **Abbreviations:** AD: Alzheimer’s disease; CeVD: cerebrovascular disease; VaD: vascular dementia; HC: healthy control; DTI: diffusion tensor imaging; FW: free-water; WM: white matter; WMH: white matter hyperintensity.

**
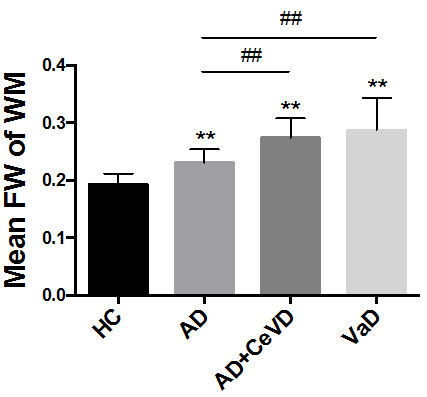
**

**Figure S2. The mean WM FW values differences between groups.** There was increased FW in the AD with and without CeVD and VaD patients compared with the HC (p = 4.6 x 10^-13^, p = 4.8 x 10^-5^ and p = 4.5 x 10^-13^, respectively, indicated by ‘**’). ‘##’ indicates increased FW in the AD+CeVD and VaD patients compared with the AD patients (p = 1.0 x 10^-6^ and p = 1.2 x 10^-8^, respectively). **Abbreviations:** AD: Alzheimer’s disease; CeVD: cerebrovascular disease; VaD: vascular dementia; HC: healthy control; DTI: diffusion tensor imaging; FW: free-water; WMH: white matter hyperintensity.


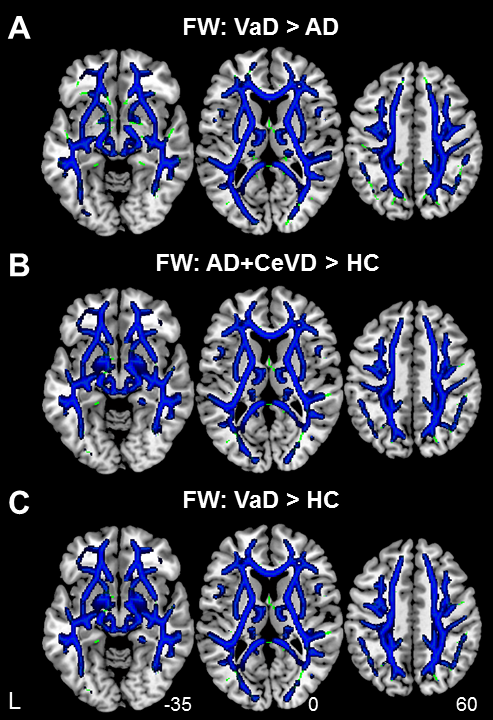


**Figure S3. Abnormal FW compartments in dementia patients.** The WM skeleton is presented in the green color. **(A)** The VaD patients exhibited a greater FW increase than the AD patients (blue color). **(B)** & **(C)** A global increase in FW values (blue color) was identified in the AD+CeVD and VaD patients. All of the results are reported at the threshold of p < 0.01, TFCE and FWE corrected. **Abbreviations:** AD: Alzheimer’s disease; CeVD: cerebrovascular disease; VaD: vascular dementia; HC: healthy control; DTI: diffusion tensor imaging; FW: free-water; WM: white matter; TFCE: threshold-free cluster enhancement; FWE: family-wise error.


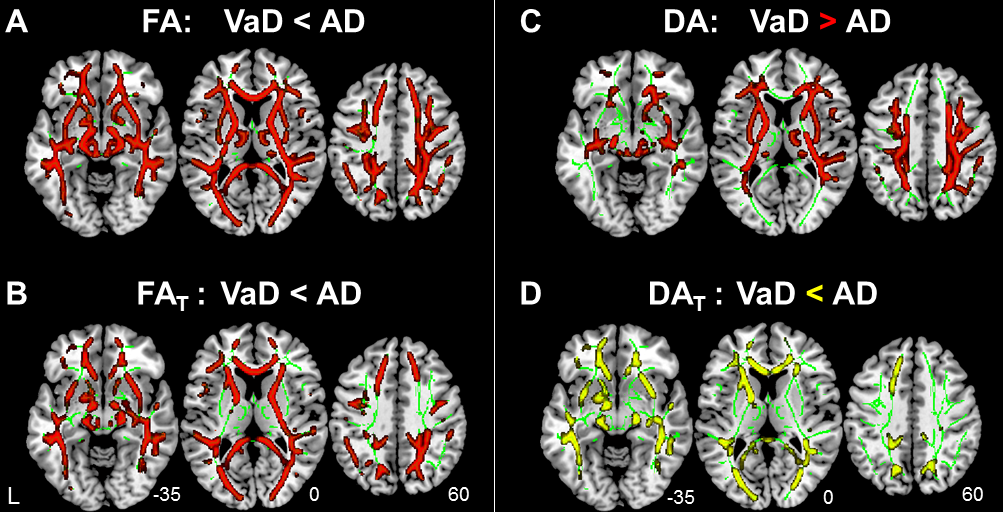


**Figure S4. Comparisons between the DTI indices and FW-corrected indices in the VaD patients. (A)** The VaD patients exhibited a greater fractional anisotropy (FA) reduction (red color) compared with the AD patients. **(B)** The FW-corrected FA_T_ group differences were less extensive and spared the subcortical and brainstem regions. The WM skeleton is highlighted in the green color. **(C)** Based on the DTI, the VaD patients exhibited a greater DA increase (red color) compared with the AD patients. **(D)** Following FW elimination, a focal cortical DA_T_ reduction was identified (yellow color). The WM skeleton is highlighted in the green color. All of the results are reported at the threshold of p < 0.01, TFCE and FWE corrected. **Abbreviations:** AD: Alzheimer’s disease; VaD: vascular dementia; HC: healthy control; DTI: diffusion tensor imaging; FW: free-water; WM: white matter; FA: fractional anisotropy; DA: axial diffusivity; FA_T_: FW-corrected FA; DA_T_: FW-corrected DA; TFCE: threshold-free cluster enhancement; FWE: family-wise error.


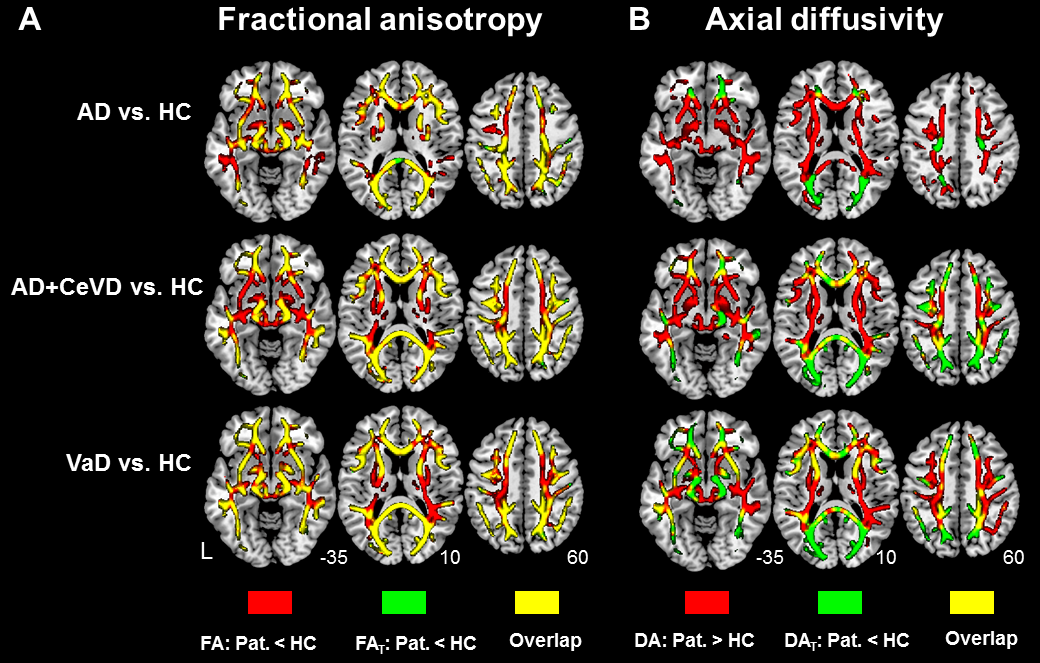


**Figure S5.** **Comparisons between DTI indices and FW-corrected tissue compartment indices in the dementia subtypes. (A)** Reduced FA or FA_T_ in all of the dementia subtypes compared to the HC was identified with or without FW corrections. The FA_T_ group difference pattern was less extensive (sparing the subcortical and brainstem regions) than the FA pattern. The regions showing reductions in both FA and FA_T_ are highlighted in yellow (overlap). The regions with FA reductions only (based on the original tensor model) are highlighted in red, whereas the FW-corrected FA_T_ group differences only are highlighted in green. **(B)** In contrast, the group difference pattern based on DA and DA_T_ were in opposite directions. Based on the original tensor indices, all of the dementia subtypes had both cortical and subcortical DA enhancements compared with the HC (red color). Following FW elimination, a focal cortical DA_T_ reduction was identified in the dementia patients (green color). All of the results are reported at the threshold of p < 0.01, TFCE and FWE corrected. **Abbreviations:** AD: Alzheimer’s disease; CeVD: cerebrovascular disease; VaD: vascular dementia; HC: healthy control; DTI: diffusion tensor imaging; FW: free-water; WM: white matter; FA: fractional anisotropy; DA: axial diffusivity; FA_T_: FW-corrected FA; DA_T_: FW-corrected DA; TFCE: threshold-free cluster enhancement; FWE: family-wise error.


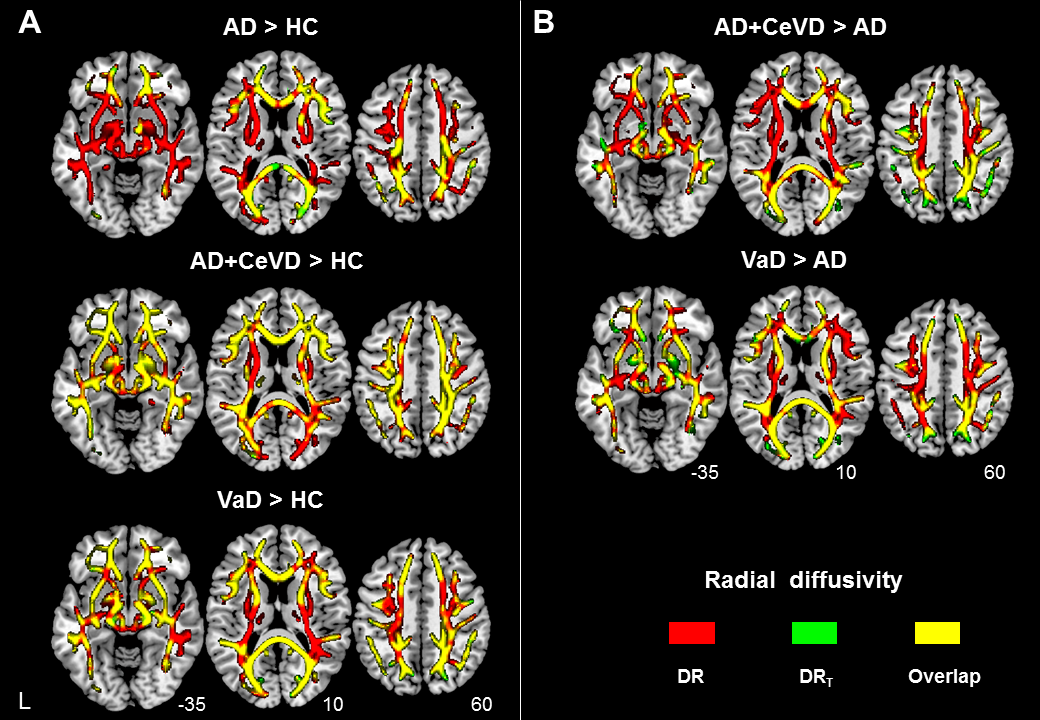


**Figure S6. Comparisons between the DR and FW corrected DR_T_ in the different dementia subtypes and HC.** **(A)** Increased DR in all of the dementia subtypes was identified with or without FW corrections. **(B)** Increased DR (DR_T_) in the AD+CeVD and VaD patients compared with the AD patients was identified with or without FW corrections, although the DR_T_ pattern was less extensive (sparing the subcortical and brainstem regions) than the DR pattern. The regions showing group differences in both DR and DR_T_ are highlighted in yellow (overlap). The regions with DR differences only (based on the original tensor model) are highlighted in red, whereas the FW-corrected DR_T_ group differences only are highlighted in green. All of the results are reported at the threshold of p < 0.01, TFCE and FWE corrected. **Abbreviations:** AD: Alzheimer’s disease; CeVD: cerebrovascular disease; VaD: vascular dementia; HC: healthy control; DTI: diffusion tensor imaging; FW: free-water; WM: white matter; DR: radial diffusivity; DR_T_: FW-corrected DR; TFCE: threshold-free cluster enhancement; FWE: family-wise error.


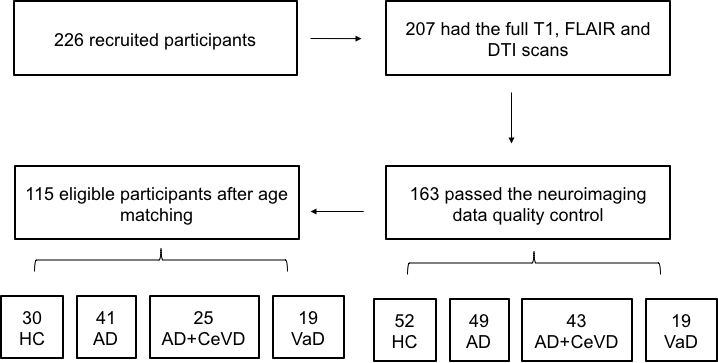


**Figure S7. Participants recruitment and selection.** Out of the 226 eligible participants recruited, 19 participants did not have full T1, DTI, and FLAIR scans, and 44 participants did not pass neuroimaging data quality control. After age and gender matching of the remaining 163 participants, a subset of 115 subjects was included for analyses. **Abbreviations:** AD: Alzheimer’s disease; CeVD: cerebrovascular disease; VaD: vascular dementia; HC: healthy control; DTI: diffusion tensor imaging; FLAIR: fluid attenuated inversion recovery.

**References**

1. Narasimhalu K, Ang S, De Silva DA, Wong MC, Chang HM, Chia KS, Auchus AP, Chen C: **Severity of CIND and MCI predict incidence of dementia in an ischemic stroke cohort.** *Neurology* 2009, **73:**1866-1872.

2. Hilal S, Chai YL, Ikram MK, Elangovan S, Yeow TB, Xin X, Chong JY, Venketasubramanian N, Richards AM, Chong JP, et al: **Markers of cardiac dysfunction in cognitive impairment and dementia.** *Medicine* 2015, **94:**e297.

3. Ong Y-TT, Hilal S, Cheung CY, Venketasubramanian N, Niessen WJ, Vrooman H, Anuar AR, Chew M, Chen C, Wong TY, Ikram MK: **Retinal neurodegeneration on optical coherence tomography and cerebral atrophy.** *Neurosci Lett* 2015, **584:**12-16.

4. Hilal S, Tan CS, Xin S, Amin SM, Wong TY, Chen C, Venketasubramanian N, Ikram MK: **Prevalence of cognitive impairment and dementia in Malays - Epidemiology of Dementia in Singapore Study.** *Curr Alzheimer Res* 2015.
